# The Risk of Disordered Eating in Fitness Club Members—A Cross-Sectional Study

**DOI:** 10.3390/sports12120343

**Published:** 2024-12-12

**Authors:** Christina Gjestvang, Therese F. Mathisen, Solfrid Bratland-Sanda, Lene A. H. Haakstad

**Affiliations:** 1 Department of Sports Medicine, Norwegian School of Sports Sciences, Ullevål Stadion, P.O. Box 4014, NO-0806 Oslo, Norway; lahaakstad@nih.no; 2 Faculty of Health, Welfare and Organization, Østfold University College, P.O. Box 700, NO-1757 Halden, Norway; therese.f.mathisen@hiof.no; 3 Department of Sports, Physical Education and Outdoor Studies, University of Southeastern Norway, Kjølnes Ring 56, NO-3918 Porsgrunn, Norway; solfrid.bratland-sanda@usn.no

**Keywords:** BMI, body appreciation, disordered eating, exercise behavior, exercise motivation, fitness club members

## Abstract

Fitness clubs may be environments where abnormal eating behaviors and excessive exercise are socially accepted, potentially putting individuals at risk for disordered eating (DE). This study examined the DE risk prevalence among gym members, comparing body appreciation, exercise motivation, frequency, BMI, and age across DE risk levels, and assessed the associated factors. A sample of 232 gym members (age: 39.6 ± 13.7) completed an online survey measuring DE risk (Eating Disorder Screen for Primary Care (ESP)), body appreciation (Body Appreciation Scale version 2), and exercise motivation (Behavioral Regulation in Exercise Questionnaire-2). One out of five (19.4%) were at risk of DE, while 15.5% and 11.6% reported having secretive eating behaviors and a history of eating disorders, respectively. Body weight impacted self-perception for 62.5% of the respondents. Those at DE risk had a higher mean BMI (26.23 ± 4.36 vs. 24.68 ± 3.61, *p* = 0.032) with a higher proportion of those with a BMI of 25–29.9 (46.67% vs. 29.41%, *p* = 0.027) compared to non-at-risk individuals. DE risk was associated with lower body appreciation (3.00 ± 3.60 versus 4.00 ± 3.70, *p* = ≤0.001), with body appreciation being the only factor associated with DE risk (OR = 0.24, 95% CI = 0.15, 0.39, *p* = ≤0.001).

## 1. Introduction

Fitness clubs are popular exercise arenas, with approximately 185 million members and 210,000 clubs worldwide [1]. However, fitness clubs are environments with marketing strategies and exercise concepts where appearance and leanness are emphasized [2]. This emphasis may not only lead to body appearance pressure and body figure idealization, which are frequently reported among fitness instructors [3,4], but also contribute to frustration and mental health disorders among exercising individuals. Research has shown that dissatisfaction with one’s body and the pressure to conform to slimness ideals are associated with heightened psychological distress, including anxiety and depression [5,6]. This may increase the risk of developing disordered eating (DE), capturing all dimensions of severe or less severe eating symptomatology such as binge eating, fasting, vomiting, the use of dieting supplements, excessive exercise, and increased levels of body dissatisfaction [7]. At worst, DE may require further investigation of the actual presence of an eating disorder [8,9]. Coinciding with this, the symptoms of DE have been frequently reported in fitness instructors and personal trainers (17% to 59%) [3,4]. In contrast to this is body appreciation, which refers to body acceptance and respect as well as the rejection of media-promoted appearance ideals [10,11], which has been found to be a protective factor against DE, as it negatively correlates with body dissatisfaction, body figure idealization, and experience of body appearance pressure [3,12,13].

It is reasonable to assume that appearance valuation and the risk of DE are prominent among gym members, as studies have found greater concern about appearance in this population compared with members of sports clubs [14,15]. Counterproductive figure idealization may create less favorable exercise motives, lead to unhealthy behaviors, and thereby increase the risk of DE [3,16]. Trott et al. (2021a) found that 30% of gym members reported body dysmorphic disorder [17]. This mental health condition is characterized by an individual’s excessive preoccupation with perceived flaws in their appearance [18]. Furthermore, these authors found that 77% of the members who reported DE also reported body dysmorphic disorder. We previously showed that exercise motives such as “looking more attractive” or “enhancing my appearance” were frequently reported (42% to 58%) among new gym members [17]. In addition, the numbers reporting such exercise motives increased throughout the first year as a member [19].

Although there are high levels of body dissatisfaction in the general population [20], few studies have investigated this among members of fitness clubs [21,22]. Fitness clubs may be environments where deviation from normal eating behavior and participation in excessive exercise are socially accepted behaviors [23]. The same authors have found that individuals who exercise excessively are encouraged to maintain this behavior by friends and family. The combination of high exercise frequency, body dissatisfaction, and societal expectations may contribute to an increased risk of DE in fitness club members [24,25,26]. Despite this, there is limited knowledge about the risk of DE among members of fitness clubs, with only a handful of studies in this field [17,27,28,29]. One study among 128 gym members found a DE prevalence of 11% [27]. In comparison, Stapleton et al. (2016), recruiting exclusively men, found that the risk of DE was higher in those exercising at gyms than in those not exercising at a gym [28]. Similarly, Mangweth-Matzek et al. (2022), also in a study among men only, found that those using a fitness club had more than three times higher rates of DE than those who were not members of a gym (5% versus 1.5%) [29]. These studies did not include women, highlighting the need for further research to explore sex differences in DE risk among fitness club members. In addition, given the potential severity of DE, further research is important to comprehensively understand the possible risk factors and protective elements unique to this specific activity setting.

Since high levels of body dissatisfaction may increase the risk of DE [30], it is essential to examine this factor in relation to the risk of DE among gym members to increase the understanding of the complex interplay between body dissatisfaction and DE. Furthermore, body dissatisfaction is relatively common in those classified as overweight/obese (body mass index (BMI) ≥ 25) [31,32]. Individuals with a high BMI and low levels of body appreciation are at higher risk of DE than individuals of normal weight [32,33]. As such, a high BMI is an important factor that might increase the risk of DE. However, body dissatisfaction may also occur among individuals classified as normal weight (BMI ≤ 25), particularly in women, who often desire a lower BMI despite being within a healthy weight range [31]. Furthermore, since DE is most prevalent during late adolescence and young adulthood, research tends to under-represent adults of all ages [34,35]. Therefore, it is essential to improve our understanding of how age is related to the risk of DE. Lastly, motivational regulation of exercise may predict dysfunctional exercise behavior such as excessive exercise [36]. Exercise is autonomous when undertaken because of its value in itself or because it is an important part of an individual’s identity. In contrast, exercise is controlled when it is initiated due to a sense of external or internal pressure [37]. While no studies have investigated this among fitness club members, more controlled forms of motivation have been found to predict body dissatisfaction and dysfunctional exercise among fitness instructors [38].

The COVID-19 pandemic significantly increased the risk of developing DE among active individuals through various mechanisms, including reduced physical activity due to quarantine measures, increased psychological distress from social isolation, and altered eating habits driven by emotional coping strategies [39,40]. In addition, we believe that disturbances in exercise routines and limited access to exercise facilities, coupled with social isolation, increased the risk of DE among fitness club members. For instance, a study of British fitness club members found an increase in the proportion of people at risk of DE following the post-COVID-19 lockdown, indicating a direct link between pandemic conditions and heightened DE risk [41]. This highlights the need for further research to understand the specific impact of such disruptions on fitness club members.

This cross-sectional study aimed to report the frequency of the risk of DE in fitness club members; compare body appreciation, exercise motivation, exercise frequency, BMI, and age between those who reported risk of DE with those who did not; and evaluate the factors associated with the risk of DE. We hypothesized the following: (1) fitness club members would exhibit a notable prevalence of a risk for DE, similar to trends observed in fitness instructors and (2) fitness club members at risk of DE would demonstrate lower body appreciation, more controlled exercise motivation, higher exercise frequency, and higher BMI compared to those not at risk.

## 2. Materials and Methods

This was a secondary analysis of data collected as a part of a cross-sectional study, aiming to compare differences in membership characteristics, including background variables, exercise motivation, and social support between members from fitness clubs with three different business models [42]. Members of selected fitness club chains in Oslo, Norway, were recruited between August 2020 and November 2020, via an email invitation from their respective gym. The chief executive officers (CEOs) distributed the email invitation to their members. Unfortunately, the CEOs did not provide the actual number of invited members, and the number of recruited participants was also not satisfactory due to the outbreak of COVID-19 and the closing of fitness clubs. Thus, recruitment was expanded to social media as an advertisement on Facebook.

**Figure 1:**
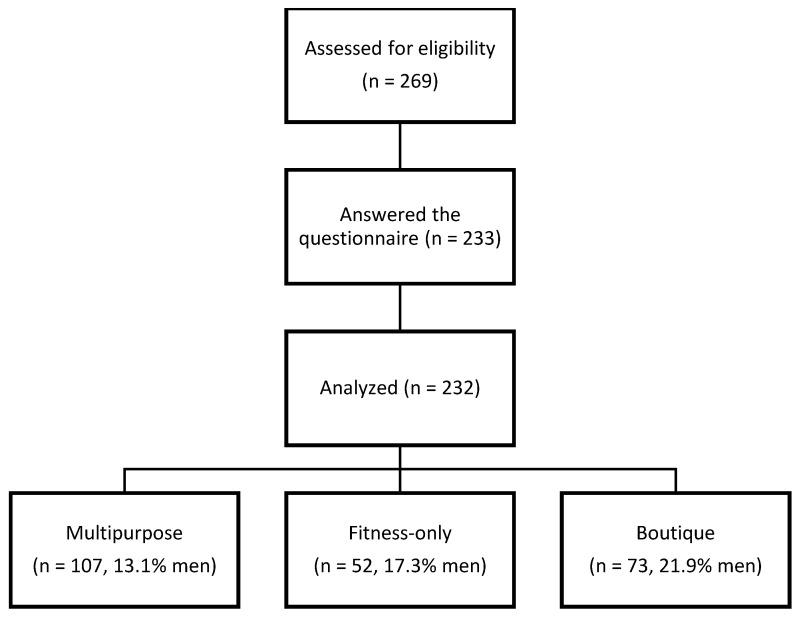
Flowchart of the participants.

The eligibility criteria were as follows: ≥18 years and member of one of three different fitness club segments. The three fitness club segments were multipurpose (e.g., resistance and cardiovascular exercise rooms, group exercise classes, and a wide range of exercise concepts, middle to high membership fee), fitness-only (e.g., resistance and cardiovascular exercise rooms for individual exercise, low membership fee), or boutique fitness clubs (e.g., one or two specialized exercise concepts, high membership fee). A total of 269 members agreed to participate in this study, of whom 233 completed the questionnaire. One questionnaire response was incomplete and therefore not used for further analysis. Hence, 232 participants (86.2% of responders, multipurpose n = 107, fitness-only n = 52, boutique n = 73) were included in the present dataset, of whom 39 (16.8%) were men. The flow of participants is shown in Figure 1.

### 2.1. Data Collection

An electronic questionnaire (SurveyXact 8.2) was used to answer the present study’s aims.

The risk of DE was assessed by a short screening instrument The Eating Disorder Screen for Primary Care (ESP), which consists of four questions with the response options “Yes” and “No” [43]. The ESP has been shown to be a valid instrument when compared with the SCOFF Questionnaire Screen for Eating Disorders [43,44]. Responses indicative of the risk of DE were defined as answering “No” to question 1 (“Are you satisfied with your eating patterns?”) and “Yes” to questions 2–4 (e.g., Do you currently suffer with or have you ever suffered in the past with an eating disorder?”), representing the need for further investigation of the actual presence of an eating disorder [43]. This cutoff was found to have a sensitivity of 100% with a corresponding specificity of 71% [43].

To evaluate internal consistency, reflecting the level of agreement among the items in the ESP, we calculated Cronbach’s α [45]. This statistic provides a value ranging from 0 to 1, with higher values indicating greater consistency in participants’ responses across the set of questions. In the current study, as expected, Cronbach’s α was low (0.21) for the ESP due to the limited number of items and should be interpreted with care [46]. In light of the low Cronbach’s α on the ESP, we conducted a thorough analysis to understand the potential sources of this low value [46]. First, we conducted Bartlett’s test of sphericity, along with the Kaiser–Meyer–Olkin (KMO) measure, to assess the suitability of our data for factor analysis [47]. A value of 0.58 (*p* = ≤0.001) indicated that factor analysis would be appropriate for our data [47,48]. Furthermore, a factor analysis with Varimax rotation was performed on the five questionnaire items to explore the structure of the underlying factor related to eating behaviors among our sample [47,48]. The analysis revealed two factors based on eigenvalues greater than 1, accounting for a total of 36.3% of the variance. The first factor, which we labeled “Eating behavior”, included items related to satisfaction with eating patterns (“Are you satisfied with your eating patterns?”) and secret eating behaviors (“Do you ever eat in secret?”), showing loadings of −0.50 and 0.66, respectively. The second factor encompassed items regarding a history of eating disorders (“Have any members of your family suffered with an eating disorder?” and “Do you currently suffer with, or have you ever suffered in the past with an eating disorder?”), with loadings of 0.69 and 0.60, respectively. Communalities for these items ranged from 0.18 to 0.48, indicating a moderate explanation of the variance by the extracted factors. Therefore, the ESP distinguished between eating behaviors and a history of eating disorders, a possible explanation for the low Cronbach’s α for the survey. The Cronbach’s α for the new subscales were 0.94 and 0.57 for “Eating behaviors” and “History of eating disorders”, respectively. It was not feasible to assess the internal consistency for the final question of the five (“Does your weight affect the way you feel about yourself?”) due to its singular-item nature.

The Body Appreciation Scale version 2 (BAS-2) was used to assess body appreciation. The BAS-2 encompass ten statements (e.g., “I appreciate the different and unique characteristics of my body”) in which the individuals rate the acceptance of, favorable opinions toward, and respect for their bodies on a 5-point scale [10]. A total score was calculated by adding scores from each statement, divided by the number of statements, with a higher average score indicating a higher level of body appreciation. BAS-2 has been shown to have good validity and reliability as well as test–retest reliability and internal consistency (Cronbach’s α > 0.65) based on sex and age [10]. In our study, the internal consistency of BAS-2 was high, as determined by the Cronbach’s α: 0.96.

Measurement of exercise motivation was based on The Behavioral Regulation in Exercise Questionnaire-2 (BREQ-2), which includes 19 statements (e.g., “I get pleasure and satisfaction from participating in exercise”), where the individuals rate the significance of each statement as a personal motive to engage or not engage in exercise on a 5-point scale [49]. The statements were divided into five subscales (intrinsic regulation, identified regulation, introjected regulation, external regulation, and amotivation), and a sum score (from 0 to 4) for each subscale was calculated by adding scores from each statement, divided by the number of statements. BREQ-2 had acceptable internal consistency for all five subscales (Cronbach’s α > 0.7) [49,50]. In this study, the internal consistency of the entire BREQ-2, as determined by Cronbach’s α, was 0.64. The BREQ-2 also gives a relative autonomy index (RAI), a direct measure of motivational autonomy (the degree to which an individual feels self-determined), proposed by Ryan and Deci (2000). This index was calculated by applying a weighting to each BREQ-2 subscale and then summing these weighted scores. The possible RAI values range from −24 to 20, where a high positive RAI score indicates greater relative autonomy, and a low negative RAI score indicates more controlled regulation [51].

To obtain exercise frequency, the participants were asked to report their average weekly exercise frequency (days/week) with the question: “On average, how many days per week do you exercise?” We also obtained data on age, sex, body weight, and height. Self-reported weight and height enabled the computation of BMI. The weight in kilograms was divided by the square of the height in meters (kg/m^2^). All questions were close-ended, and the survey took 20 min to complete.

In the preparation of this manuscript, artificial intelligence (AI) technology was utilized to assist with drafting and editing text. Specifically, OpenAI’s ChatGPT, version GPT-4, was employed to refine the clarity and coherence of the narrative. All content generated using AI was subsequently reviewed and revised by the authors to ensure accuracy, relevance, and alignment with this study’s objectives.

### 2.2. Statistics

All statistical analyses were conducted using SPSS Software V. 24 (IBM Corp. Released 2016. IBM SPSS Statistics for Windows, Armonk, NY, USA: IBM Corp). To compare background characteristics, and body appreciation, exercise motivation, exercise frequency, BMI, and age between those who reported a risk of DE and those who did not, a chi-square test or an independent t-test was used, as appropriate. Spearman’s rho was further used to examine the correlations between the risk of DE and sex, body appreciation, exercise motivation, exercise frequency, BMI, and age. We also conducted a binomial logistic regression with the risk of DE as the dependent variable, adjusting for significant variables identified by Spearman’s rho (BAS-2 total score and BMI). The results are presented as frequencies (n) and percentages, means with standard deviations (SDs) or medians with ranges, mean differences, and effect sizes (Hedge’s g) for background characteristics and comparison analyses. Hedge’s g is a standardized measure that quantifies the magnitude of differences between groups, particularly well-suited for smaller sample sizes as it provides a bias-corrected estimate compared to Cohen’s d [52]. Furthermore, the correlation coefficient (r), odds ratio (OR), and 95% CI for odds ratio were calculated for Spearman’s rho and the binomial regression. The effect size for differences in background characteristics and comparison between those who reported a risk of DE and those who did not was categorized as small (0.15), medium (0.40), and large (0.75) [52]. The level of significance was set at *p* ≤ 0.05.

## 3. Results

The background characteristics of the participants are shown in Table 1. Age ranged from 23 to 80 and 18 to 78 years in men and women, respectively. Furthermore, the proportion in age groups were as follows: 18–25 years (16.4%), 26–39 years (38.4%), 40–59 years (35.3%), and >60 years (9.9%). The majority (76.8%) had been a member of a fitness club for more than 1 year (1 to 5 years: 41.2%, >5 years: 18.0%, >10 years: 17.6%) and exercised 4.22 ± 1.50 days per week. More data on background and health factors as well as exercise behavior of the participants have been described previously [42,53].

Of all the participants, 19.4% (n = 45) were found to be at risk of DE (ESP score ≥ 2, women 19.17% (n = 37), men 20.51% (n = 8), *p* = 0.858). Furthermore, 15.5% (n = 36) of the participants reported having secretive eating behaviors, and 11.6% (n = 27) indicated having a history of eating disorders. Six out of ten (62.5%, n = 145) stated that their body weight affected their self-perception.

There was a significant difference between members at a high risk and members at a low risk of DE in the BAS-2 total score and BMI, with those at risk of DE having a higher BMI (26.23 ± 4.36 versus 24.68 ± 3.61, Hedge’s g = 0.41, *p* = 0.032) and a lower total BAS-2 score (3.00 ± 3.60 versus 4.00 ± 3.70, d = 0.27, *p* = <0.001) than those not at risk. Also, compared with those at no risk of DE, those at risk of DE were more likely to be overweight (BMI 25–29.9: 46.67% versus 29.41%, *p* = 0.027) than obese (BMI ≥ 30: 13.33% versus 8.56%, *p* = 0.326). There were no differences in motivational regulation in exercise, relative autonomy index, exercise frequency, or age (Table 2).

Spearman’s rho revealed that the total BAS-2 score (r = −0.45), *p* = ≤0.001) and BMI (r = 0.17, *p* = 0.009) were significantly associated with the risk of DE, with a moderate negative correlation (total BAS-2 score) and a weak positive correlation (BMI). Furthermore, only the total BAS-2 score was statistically significant in the binomial logistic regression (OR = 0.24, 95% CI for OR: 0.15, 0.39, *p* = <0.001) (Table 3). A higher total BAS-2 score (reporting higher body appreciation) was associated with a reduced likelihood of being at risk of DE (OR = 0.24).

## 4. Discussion

Our main finding was that one out of five participants reported being at risk of DE, with no sex differences observed. Thus, our first hypothesis, which posited a notable prevalence of DE risk among fitness club members, similar to trends observed in fitness instructors, was not supported. Our second hypothesis, that fitness club members at risk of DE would demonstrate lower body appreciation, was supported, as higher body appreciation was associated with a lower risk of DE. Further investigation is needed to explore the other predicted factors, including exercise motivation, exercise frequency, and BMI concerning the risk of DE.

### 4.1. The Risk of DE

Approximately 20% of the participants reported being at risk of DE. Our study was conducted during the COVID-19 pandemic, and a large amount of the data from countries such as Norway showed a massive increase in mental health challenges, including a rise in eating disorders [54,55]. The COVID-19 pandemic led to significant disruptions in exercise routines and limited access to exercise facilities for fitness club members, in addition to social isolation. These changes were suggested to exacerbate concerns about weight and body shape, potentially increasing the risk of developing eating disorders [56]. Although physical activity has been found to be a protective factor for the development of mental health symptoms when people stayed at home [57], we cannot exclude the possibility that the COVID-19 pandemic increased the risk of DE among our participants. Supporting this, a study among British fitness club members found an increase in the proportion at risk of DE following the post-COVID-19 lockdown, from 25.5% before to 27.3% after lockdown [41].

Given the high prevalence of risk of DE in fitness instructors [3,4], we hypothesized a similar trend among fitness club members. However, our participants reported a nearly 30% lower risk of DE than fitness instructors. Also, our analysis revealed that a minority of the participants had secretive eating behaviors or a history of eating disorders. Explanations for the different results may be that fitness instructors believe that they should be slim, athletic, and close to the current Western body ideal since their appearance is a condition of professional success [58]. Furthermore, it may be that this profession attracts individuals already preoccupied with body concerns, similar to what has been found in leanness sports [59]. An overemphasis on appearance and pressure from fitness instructors may create an ego-involving climate, which may discourage members from participating in exercise. Such environments, which prioritize performance outcomes and comparisons, have been linked to reduced motivation and adverse effects on psychological well-being, including anxiety and diminished self-esteem [60,61]. Research indicates that fostering a positive, task-involving, and caring climate is crucial in promoting long-term engagement and mental health benefits among fitness club members [61,62].

### 4.2. Factors Associated with the Risk of DE

Our findings might add to the hypothesis that individuals prone to counterproductive figure idealization are more likely to join exercise arenas such as fitness clubs. For instance, a significant number of participants in our study reported that their body weight influenced their self-perception. Furthermore, in the qualitative study by Riseth et al. (2019), fear of becoming overweight and a desire to become more muscular were reasons to use the fitness club among long-term members. These members also highlighted that their focus on physical appearance was due to the increased emphasis in society on being thinner and fit [15]. In addition, it has been shown that adolescent boys with weight and shape concerns more frequently exercised at fitness clubs compared with adolescent boys participating in sports clubs [63]. We have also previously reported that half of fitness club members reported appearance as a reason for exercising [19,64]. Yet, the health benefits of exercise was at the top of the list for most individuals [15,19].

The fitness club environment has previously been identified as an arena in which body figure appearance is idealized [3,12,63]. Fitness clubs, with their numerous full-length mirrors and posters idealizing toned and athletic bodies, provide the opportunity for direct comparison with others [21]. This context may increase concerns about personal body weight and health, as well as body appearance pressure, regardless of the admired sex-specific body figure ideal. Based on the finding that 20% of our participants were at risk of DE, we highlight the need for ethical reflections in the marketing strategies and exercise concepts at fitness clubs, focusing on appearance and body weight. Furthermore, our findings also support the importance of fitness club employees’ knowledge about DE, how to identify DE, and how to manage concerns about DE. As previous studies have shown low competence in this area [65,66], it is necessary to provide education and competence development for such occupational groups.

Finding no sex differences in the risk of DE contrasts the previous literature [4,38,67,68]. Several factors could explain this discrepancy, including the disproportion of men and women participating in this study, the use of an electronic questionnaires, age, and the fitness club context, as well as differences in the measurements used to assess DE. It is important to note that only 17% of our participants were men, which may have reduced the statistical power and increased the likelihood of type II errors. Therefore, our findings should be interpreted with caution. Sex imbalances within a study may skew results and obscure the true differences between variables [69]. Furthermore, we believe that men and women might differ in their willingness to participate in online surveys, which could influence the outcomes and obscure sex differences. Hence, future studies with a more balanced sex ratio are essential for validating our findings in various contexts. In addition, in fitness clubs, where body figure appearance is often highly idealized, the environment may induce concerns over body weight and health, leading to body image pressure, regardless of prevailing sex-specific body figure ideals [3,12,63].

The specific questionnaire, ESP [43], used in our study might offer a more sex-neutral approach to detecting concerns related to food and body image in both sexes compared with other instruments (e.g., Eating Disorders Inventory [70] or Eating Disorders Examination Questionnaire [71]). These instruments may be more accurate for capturing women’s compared to men’s body appearance concerns. Hence, when using ESP among different sexes recruited from a venue in which they probably engage from similar motivations, it might be less likely to detect sex differences in the frequency of risk of DE.

Our finding that age was not related to the risk of DE aligns with those of another study reporting similar prevalence rates of DE across various age groups within the general adult population [72]. This indicates that the risk of DE remains consistent between different ages, highlighting the importance of intervention strategies that span across all age groups. However, the literature on this topic is not entirely consistent. One study found an age-related difference in the risk of DE, indicating that certain age groups may be more susceptible to DE [73]. The discrepancy in the literature could be due to differences in study design, participant characteristics, and the specific instrument used to assess the risk of DE and suggesting that age may interact with other factors influencing the risk of DE. For instance, a meta-analysis found that interactions between age, gender, and BMI significantly predicted the risk of eating disorders in medical students, while these variables alone did not [74]. Further research is essential to better understand how age influences DE, which could lead to more tailored interventions addressing the needs of different age groups.

Our finding that higher body appreciation was associated with a lower risk of DE contributes to the existing body of knowledge from studies on other populations. As such, body appreciation is an important modifiable protective factor for the development of DE and further eating disorders [20,75]. In contrast to another study showing that the risk of eating disorders was moderately positively correlated with exercise motivation regulation [38], this was not found in our study. Potential explanations for this relate to the validity of the instrument used to assess the risk of DE (ESP) and the scores on our instrument used to measure exercise motivation (BREQ-2). First, previous studies reporting that motivational regulation predicts the risk of DE or eating disorders have used more comprehensive and widely validated instruments, such as the Eating Disorders Inventory or the Eating Disorders Examination Questionnaire [70,71]. Second, the scores on the BREQ-2 in our study showed that the participants scored high on autonomous exercise motivation and low on more controlled regulation of motivation. This is in accordance with findings from a study among group fitness instructors [38] and might indicate that, in highly self-determined exercise populations, exercise motivation is less relevant as an explanatory variable for the risk of DE. It is important to note that we did not assess the specific external factors that motivated the participants. Thus, there might be differences in the importance of, e.g., weight- and appearance-related reasons for exercise compared to more health-related reasons for exercise between participants at risk and not at risk of DE.

In contrast to other highly active populations, our participants had a high mean BMI [76]. Therefore, this differs from other reports relying on the ESP screening instrument, in which inactive younger adults or fit runners attending college were studied [67,68]. Since fitness clubs offer a wide range of resistance exercise equipment, we speculate that our participants conducted high levels of resistance exercise, which is also reported as the most common exercise mode in this setting [77]. Thus, having a high muscle mass, rather than an excessive fat mass, may overestimate BMI. It has been shown that BMI does not distinguish between active and inactive individuals with dissimilar body compositions [78].

In the fitness club environment, where social pressure often forces individuals to adhere to idealized body standards, it might be hypothesized that individuals with higher muscle mass may experience pressure to further increase muscle size or definition. This pressure could lead to an increased risk of DE, manifested through excessive exercise, restrictive eating, or the misuse of supplements. For instance, research has shown that distortions of perceived body image ideals, which include concerns about fat and muscle mass, contribute to the risk of DE in men [79,80]. On the other hand, the high mean BMI observed among our participants may reflect excessive fat mass, providing reasons for a fitness club membership primarily driven by the desire to lose weight. It has been shown that body dissatisfaction is more prevalent among individuals classified as overweight or obese and, furthermore, that those with high BMI, low levels of body appreciation, and attempting to lose weight are at a heightened risk of DE [32,33].

### 4.3. Strengths and Limitations

Our study’s strongest aspect is the sample size of 232 fitness club members, which aligns with the sample sizes in similar research within this field [27,28,29]. Additionally, the diversity of our sample, including various segments and chains of fitness clubs, and the use of standardized and validated instruments, strengthen our study. However, the study findings may not be generalized due to limitations related to the sample. For instance, we did not obtain data regarding the response rate from those invited by the fitness club CEOs. In addition, the recruitment via social media may limit generalizability due to potential self-selection bias. Thus, we acknowledge that recruitment through email and Facebook advertisements and the use of electronic questionnaires has both strengths and limitations.

Additional study strengths include its feasibility and a cost-effective reach to a larger part of the population, whereas the limitations include a skewed demographic characteristic across social media platforms, e.g., Facebook users are older than those who use other social networks [81]. Furthermore, while the use of self-reported data is considered an appropriate measurement instrument for gathering information on psychological factors, we cannot rule out social desirability bias, which may influence participants to provide responses they believe are favorable rather than accurate [82]. Also, the interpretation of the survey questions may vary between participants, potentially leading to differences in how they understand and respond to the wording of the questions.

One of the primary limitations of our study is the use of only a short screening instrument for DE, with a low and varying Cronbach’s α. The Cronbach’s α of 0.21 indicates low internal consistency when considering the ESP. This low value can be attributed to several factors. First, the ESP includes items measuring distinct constructs, such as personal eating behaviors and familial history of eating disorders. Cronbach’s α assumes that all items within a scale measure the same underlying construct [83]. Hence, the diversity in the items of ESP may lead to lower alpha values. Our factor analysis also supported this by revealing two separate factors (“Eating behaviors” and “History of eating disorders”). The internal consistency within these subscales varied considerably, with alpha values of 0.94 and 0.57, respectively. This variation underscores the need for careful consideration of the constructs being measured when interpreting alpha values. However, the factor analysis provided valuable insight into the different constructs measured by the ESP. Second, the short length of the ESP may affect Cronbach’s α, and, with only five items, the ESP has limited potential to achieve high internal consistency [83]. Short scales inherently have lower reliability, as they provide fewer opportunities for items to correlate [83]. This limitation was particularly evident in our study, where the short length of the ESP contributed to the low Cronbach’s α. Also, the inability to measure internal consistency for the item “Does your weight affect the way you feel about yourself?” due to its singular nature underscores the challenges of using Cronbach’s α as the sole reliability measure of the ESP.

The sample included in this study was considered highly active (4.22 ± 1.50 exercise days per week), and the ESP has not been validated for such individuals. Thus, more widely validated instruments should be used in future studies [70,71]. However, the ESP is in line with other brief measures and has been shown to be a valid instrument when compared with the SCOFF Questionnaire Screen for Eating Disorders [43,44]. Thus, we believe the ESP is an efficient tool to help decide whether a more detailed assessment of possible DE is required. Finally, only one-fifth of our sample consisted of men, and the generalizability of our findings to all fitness club members is not justified. Additionally, recruiting a larger sample size would have allowed us to divide the analysis by sex and adjust the analyses for more covariates (such as educational level) than those included (sex, BAS-2, BREQ-2, exercise frequency, BMI, and age). Finally, a notable limitation of this study is the absence of an a priori sample size calculation, which raises concerns about the robustness of this study’s findings.

### 4.4. Practical and Scientific Implications

In our opinion, the present study provides an important understanding of the beneficial focus of body appreciation. Actors within the fitness club industry should be aware of this focus and aim to improve body appreciation among members. Fitness clubs may implement marketing that features a diverse range of body types and positive messaging focused on health rather than appearance. Offering exercise options designed to make participants feel good, rather than emphasizing “looking good”, may foster a more inclusive and welcoming community atmosphere. These efforts may encourage members to accept, respect, and hold favorable attitudes toward their bodies, reducing body objectification and fostering more sustainable, positive motivations for exercise [61].

For fitness club staff, the emphasis should similarly shift toward promoting exercise as a means of enhancing health and well-being. Creating an environment that de-emphasizes stereotypical “ideal bodies” and instead prioritizes individual progress and holistic health is essential [61]. Tailored communication strategies are also critical to challenging body image stereotypes and offering diverse representations of health and fitness. Special attention should be paid to younger participants, who often face heightened pressures regarding body image [61].

Future studies should include other instruments in addition to ESP for screening for risk of DE in fitness club members to validate the ESP in this population. Furthermore, data on the risk of DE after the COVID-19 pandemic are needed to establish risk during non-pandemic periods. The association between higher body appreciation and lower risk of DE suggests that body appreciation could be a valuable protective factor. Future studies should investigate the pathways through which body appreciation influences DE risk and how it can be effectively promoted in different populations. In addition, future research should employ longitudinal designs to better understand the causal relationships between body image and eating behaviors in fitness club contexts [61]. Including diverse populations will also broaden the understanding of these dynamics. Evaluating the long-term effects of interventions aimed at reducing body image dissatisfaction will also further enhance knowledge in this field.

## 5. Conclusions

Higher body appreciation was associated with a lower risk of DE, and one out of five participants was classified as being at risk of DE. In the fitness club industry, an increased consciousness of DE needs to be considered in marketing strategies and exercise concepts, including improving staff members’ understanding of DE. Changing the industry focus from the aesthetic to the functional aspects of the body may enhance members’ body satisfaction, hence building resilience against DE.

## Figures and Tables

**Table 1:** Background characteristics of included fitness club members.

Variables	All (n = 232)	Women (n = 193)	Men (n = 39)	Hedge’s g	*p*
	Mean ± SD	Mean ± SD	Mean ± SD		
Age (years)	39.59 ± 13.69	39.16 ± 13.44	41.77 ± 14.86	0.19	0.278
Body weight (kg)	72.77 ± 13.47	70.42 ± 12.51	84.36 ± 13.09	1.11	**≤0.001**
BMI (kg/m^2^)	24.98 ± 3.80	24.82 ± 3.81	25.80 ± 3.76	0.26	0.141
Exercise frequency (days/week)	4.22 ± 1.50	4.21 ± 1.49	4.23 ± 1.53	0.01	0.945

**Table 2:** Comparing BAS-2, motivation, exercise frequency, and BMI in individuals at risk and not at risk of DE.

Variables	Risk of DE				
	Yes (n = 45)	No (n = 187)			
	Median (Range)	Median (Range)	Mean Difference	Hedges g	*p*
Total BAS-2 score	3.00 ± 3.60	4.00 ± 3.70		0.27	**≤0.001**
	Mean ± SD	Mean ± SD	Mean difference		*p*
BREQ-2					
Intrinsic regulation	3.42 ± 0.88	3.51 ± 0.56	0.08 ± 0.32	0.14	0.407
Identified regulation	3.45 ± 0.57	3.46 ± 0.55	0.01 ± 0.02	0.02	0.864
Introjected regulation	1.65 ± 1.05	1.57 ± 1.05	0.08 ± 0.00	0.08	0.625
External regulation	0.21 ± 0.44	0.16 ± 0.34	0.04 ± 0.10	0.14	0.507
Amotivation	0.06 ± 0.33	0.04 ± 0.18	0.01 ± 0.15	0.09	0.769
RAI	14.90 ± 4.54	15.42 ± 3.17	0.52 ± 1.37	0.15	0.467
Exercise frequency	3.87 ± 1.74	4.31 ± 1.43	0.44 ± 0.31	0.29	0.076
BMI (kg/m^2^)	26.23 ± 4.36	24.68 ± 3.61	1.54 ± 0.75	0.41	**0.032**
Age (years)	39.00 ± 12.36	39.82 ± 14.01	0.81 ± 2.27	0.59	0.720
	n (%)	n (%)	Mean difference		*p*
BMI 25–29.9	21 (46.67)	55 (29.41)	34 (17.26)		**0.027**
BMI ≥ 30	6 (13.33)	16 (8.56)	10 (4.77)		0.326
Age group					0.768
18 to 25 years	6 (16.22)	31 (83.78)			
26 to 39 years	19 (21.35)	70 (78.54)			
40 to 59 years	17 (20.73)	65 (79.27)			
≥ 60 years	3 (13.04)	20 (86.96)			

**Table 3:** Binomial logistic regression analysis summary for total BAS-2 score and BMI predicting risk of DE.

	ORs	95% CI for ORs		*p*
Variable		Lower	Upper	
Total BAS-2 score	0.24	0.15	0.39	**≤0.001**
BMI	1.03	0.94	1.13	0.492
Constant	13.96			0.093

## Data Availability

The raw data supporting the conclusions of this article will be made available by the authors without undue reservation.
